# Supplementary material for: Functional BRI2-TREM2 interactions in microglia: implications for Alzheimer’s and related dementias
Source: EMBO Rep. 2024 Feb 12;25(3):23. doi: 10.1038/s44319-024-00077-x (PMC10933458; doi:10.1038/s44319-024-00077-x)
Supplement: Supplementary file 4 — Source Data Fig. 8 [file 44319_2024_77_MOESM4_ESM.zip › Source data Fig 8/Source data Fig 8A right FACS panel/20210816 Brain-Batch_Analysis_16082021135155.pdf]

BD FACSDiva 8.0.2

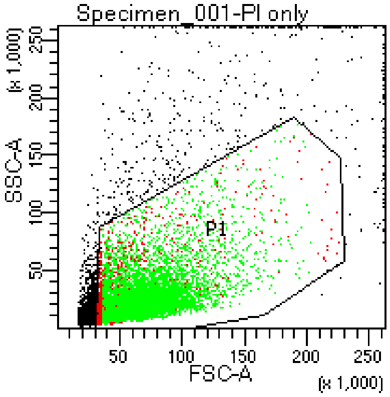

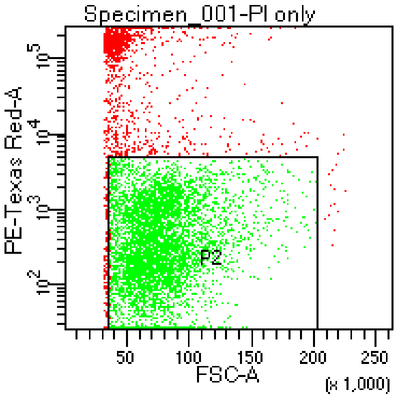

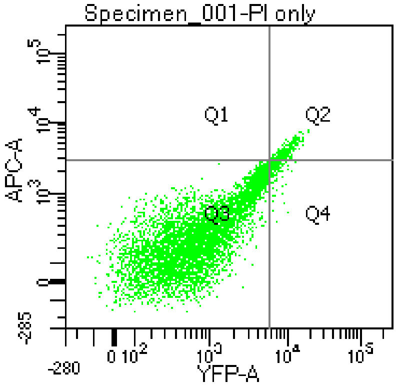

Tube: PI only

Population	#Events	%Parent	%Total
All Events	10,000	####	100.0
P1	7,664	76.6	76.6
P2	5,906	77.1	59.1
Q1	6	0.1	0.1
Q2	268	4.5	2.7
Q3	5,406	91.5	54.1
Q4	226	3.8	2.3

BD FACSDiva 8.0.2

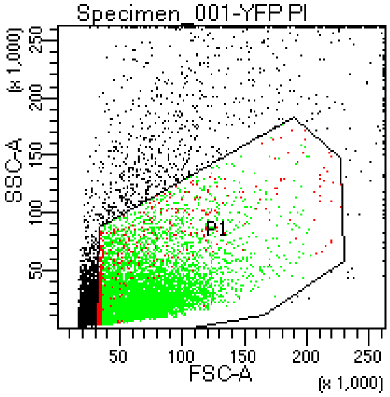

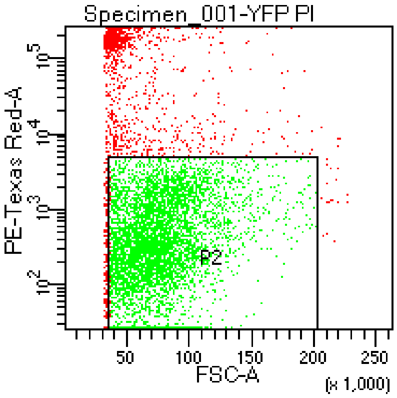

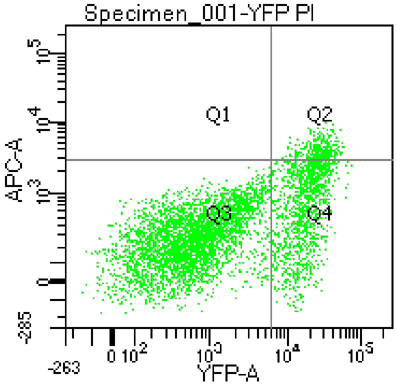

Tube: YFP PI

Population	#Events	%Parent	%Total
All Events	10,000	####	100.0
P1	6,749	67.5	67.5
P2	5,251	77.8	52.5
Q1	0	0.0	0.0
Q2	333	6.3	3.3
Q3	3,623	69.0	36.2
Q4	1,295	24.7	13.0

BD FACSDiva 8.0.2

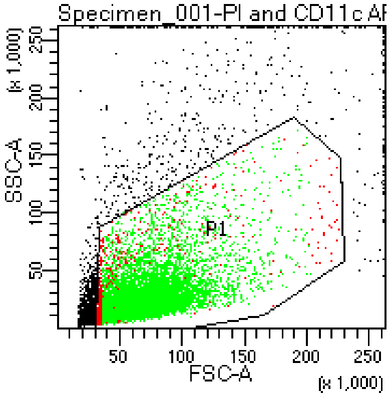

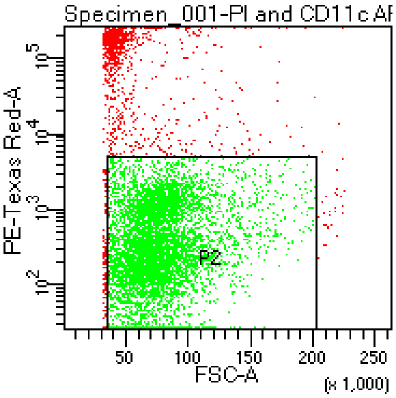

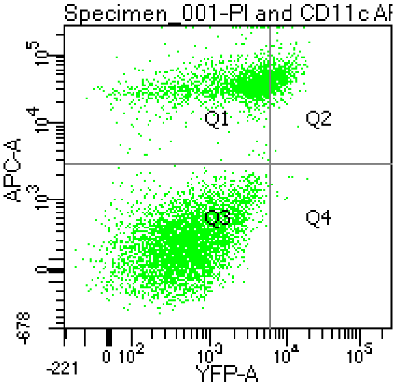

Tube: PI and CD11c APC

Population	#Events	%Parent	%Total
All Events	10,000	####	100.0
P1	7,985	79.8	79.8
P2	6,661	83.4	66.6
Q1	1,779	26.7	17.8
Q2	563	8.5	5.6
Q3	4,301	64.6	43.0
Q4	18	0.3	0.2

BD FACSDiva 8.0.2

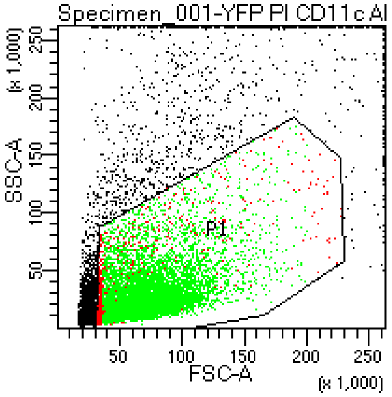

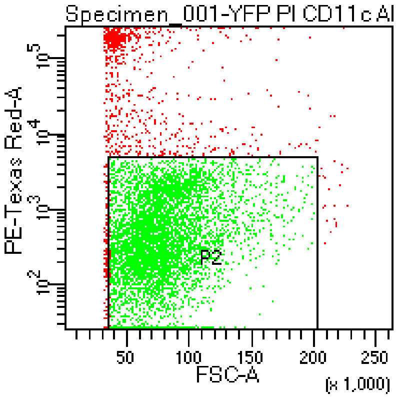

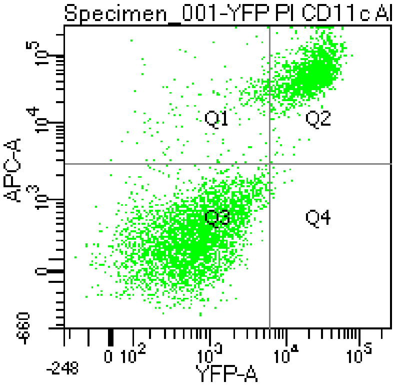

Tube: YFP PI CD11c APC

Population	#Events	%Parent	%Total
All Events	10,000	####	100.0
P1	7,087	70.9	70.9
P2	5,830	82.3	58.3
Q1	212	3.6	2.1
Q2	1,875	32.2	18.8
Q3	3,722	63.8	37.2
Q4	21	0.4	0.2
