# Supplementary material for: Functional BRI2-TREM2 interactions in microglia: implications for Alzheimer’s and related dementias
Source: EMBO Rep. 2024 Feb 12;25(3):23. doi: 10.1038/s44319-024-00077-x (PMC10933458; doi:10.1038/s44319-024-00077-x)
Supplement: Supplementary file 4 — Source Data Fig. 8 [file 44319_2024_77_MOESM4_ESM.zip › Source data Fig 8/Source data Fig 8B FACS /CF+3M_-purity.pdf]

BD FACSDiva 8.0.1

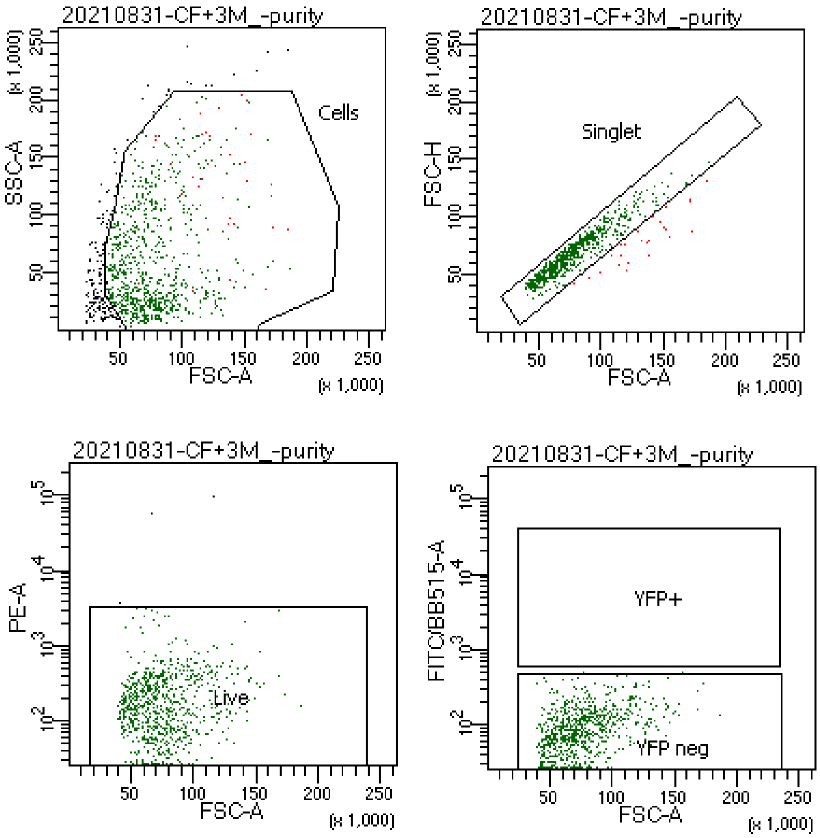

Tube: CF+3M_-purity

Population	#Events	%Parent	%Total
All Events	1,000	####	100.0
Cells	803	80.3	80.3
Singlet	772	96.1	77.2
Live	769	99.6	76.9
YFP+	0	0.0	0.0
YFP neg	769	100.0	76.9
