# Supplementary material for: Functional BRI2-TREM2 interactions in microglia: implications for Alzheimer’s and related dementias
Source: EMBO Rep. 2024 Feb 12;25(3):23. doi: 10.1038/s44319-024-00077-x (PMC10933458; doi:10.1038/s44319-024-00077-x)
Supplement: Supplementary file 4 — Source Data Fig. 8 [file 44319_2024_77_MOESM4_ESM.zip › Source data Fig 8/Source data Fig 8B FACS /20210831_CF+3M_+purity.pdf]

BD FACSDiva 8.0.1

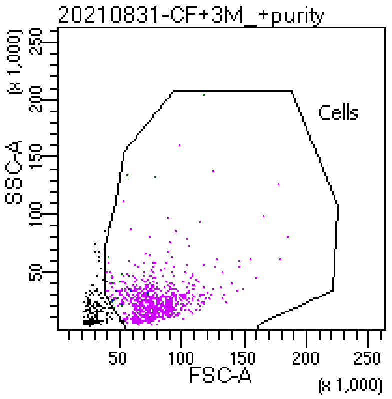

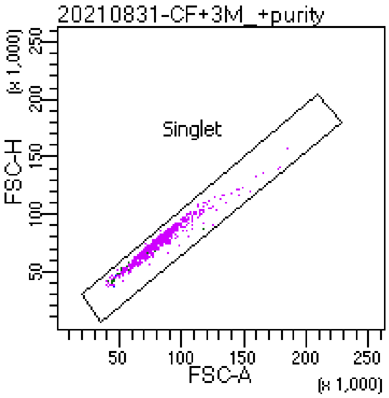

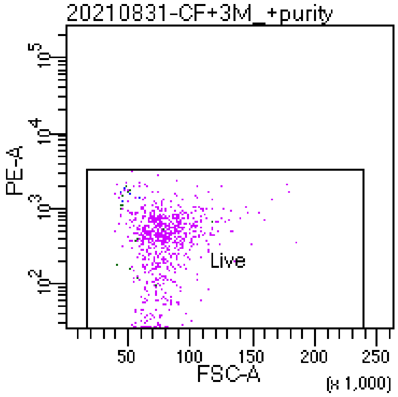

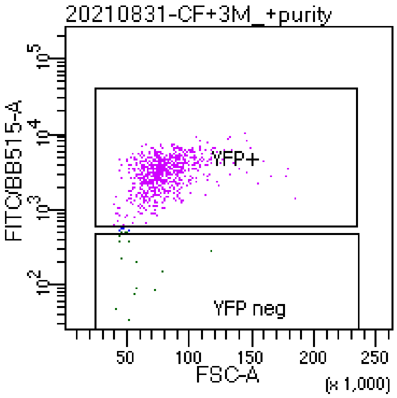

Tube: CF+3M_+purity

Population	#Events	%Parent	%Total
All Events	1,000	####	100.0
Cells	782	78.2	78.2
Singlet	782	100.0	78.2
Live	782	100.0	78.2
YFP+	761	97.3	76.1
YFP neg	15	1.9	1.5
