# Supplementary material for: Functional BRI2-TREM2 interactions in microglia: implications for Alzheimer’s and related dementias
Source: EMBO Rep. 2024 Feb 12;25(3):23. doi: 10.1038/s44319-024-00077-x (PMC10933458; doi:10.1038/s44319-024-00077-x)
Supplement: Supplementary file 13 — Expanded View Figures [file 44319_2024_77_MOESM13_ESM.pdf]

Expanded View Figures

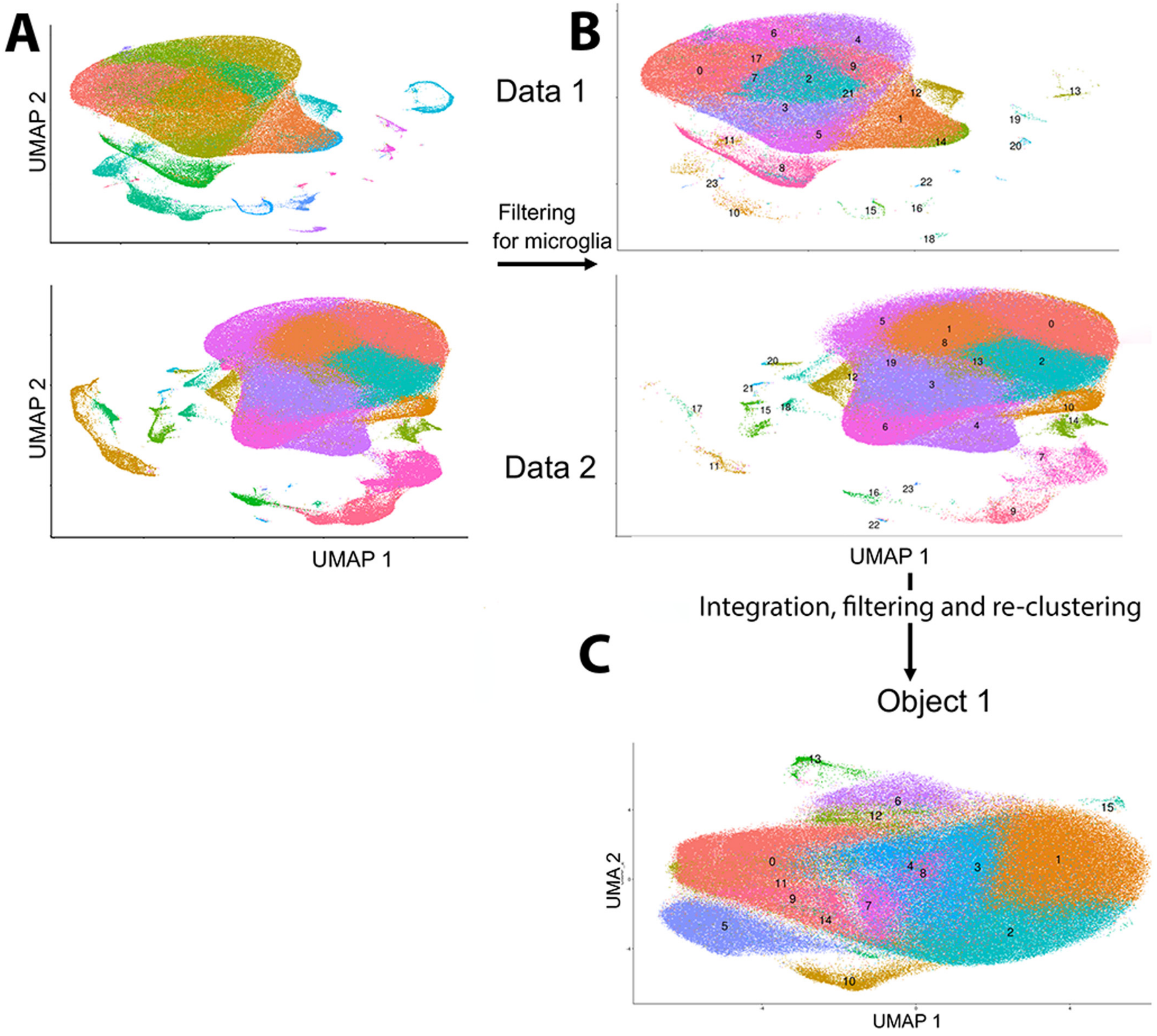

Figure EV1. UMAP of Data 1 and Data 2.

(**A**) UMAPs of objects Data 1 and Data 2 before filtering. (**B**) UMAPs of objects Data 1 and Data 2 after filtering. (**C**) UMAP of Object 1 after integration, filtering, and re-clustering, shows 16 microglia clusters. Data information: The data presented in this analysis are the result of two experiments, namely Data 1 and Data 2. To combine specific sample datasets from both Data 1 and Data 2, we employed the integration feature within the Seurat package. By utilizing the first 20 principal components, we integrated these datasets into a single entity referred to as "Object1," which encapsulates information from a total of 297,215 cells. These cells derive from: *Trem2-KO*, 1 male and 1 female; *Itm2b-KO*, 2 males and 2 females; WT controls, 1 male and 2 females; *Itm2b/Trem2-dKO*, 1 male and 1 female. The scRNAseq data are deposited at https://www.ncbi.nlm.nih.gov/geo/info/seq.html, GSE233601 to allow public access once the data are published.

© The Author(s)

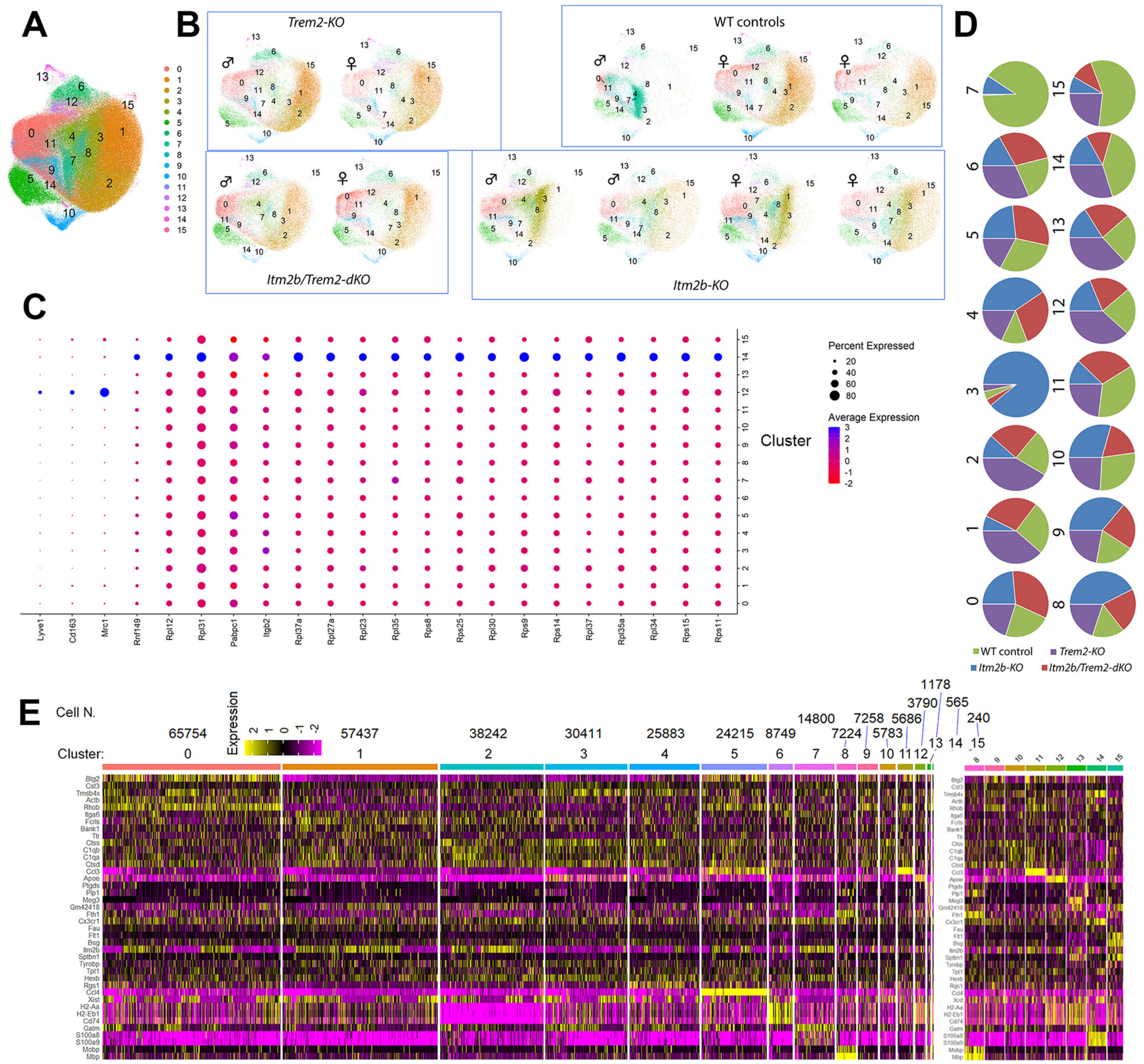

Figure EV2. Object 1 supporting information.

(**A**) UMAPs of re-clustered microglia in Object 1. (**B**) UMAPs split by individual samples. (**C**) Average scaled expression levels of selected signature genes per cluster and cluster's annotation based on expression of signature genes. (**D**) Proportional contribution of each genotype to each cluster. Cluster 3 was highly represented in *Itm2b-KO* mice, with 89% of microglia in this cluster originating from these mice. Conversely, Cluster 7 was preponderant in WT controls. However, ~93% of the cells assigned to cluster 7 derived from one WT control animal (the male WT control, as depicted in UMAP plot b). Therefore, the observed expansion of cluster 7 is attributed to animal-specific factors rather than genotype-specific factors. (**E**) Gene expression heatmap showing the top 5 enriched genes for each microglia cluster. The number of cells per cluster is denoted above the cluster. *Itm2b* is one of the top genes downregulated in cluster 3 because 89% of the cells in this cluster are from *Itm2b-KO* mice. Enlarged heatmap of Clusters 8 to 15 is also show (right) for better visibility. Data information: The data presented in this analysis are deposited at https://www.ncbi.nlm.nih.gov/geo/info/seq.html, GSE233601 to allow public access once the data are published.

 © The Author(s)

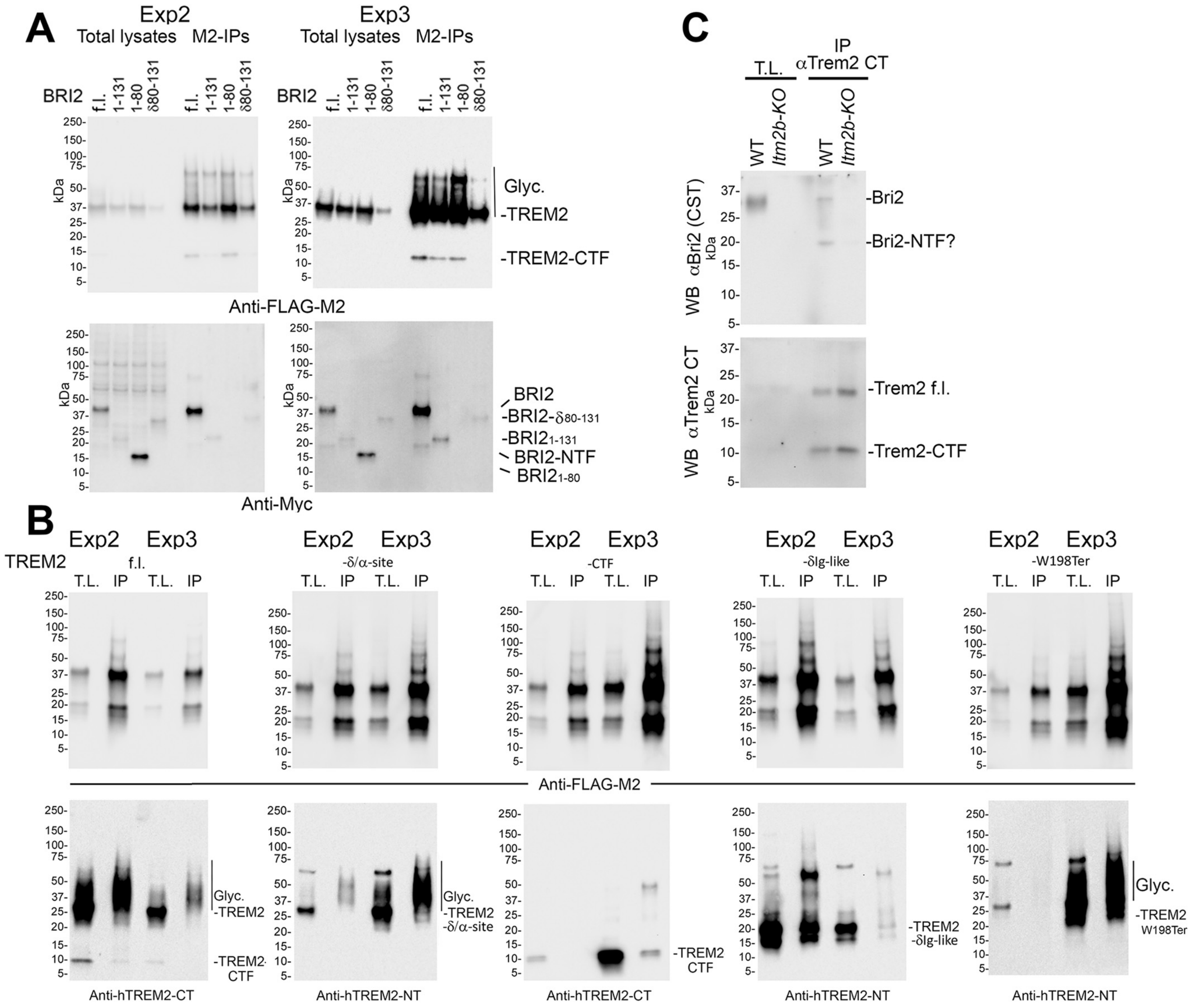

Figure EV3. Interaction between human BRI2 and TREM2 in transfected cells and co-immunoprecipitation of endogenous Bri2 and Trem2 in mouse primary macrophages.

(**A**) Western blot analysis with anti-FLAG, anti-Myc antibodies, and anti-human BRI2 antibodies of total lysates and immunoprecipitated samples (IP-M2) from transfected HEK293 cells. These experiments are biological replicates of the experiment shown in Fig. 4B. (**B**) Western blot analysis with anti-FLAG, anti-human TREM2-NT, and anti-human TREM2-CT antibodies of total lysates (T.L.) and immunoprecipitated samples (IP-M2) from transfected HEK293 cells. These experiments are biological replicates of the experiment shown in Fig. 4D. (**C**) Co-immunoprecipitation of endogenous Bri2 and Trem2 from mouse primary macrophages. Samples were deglycosylated before Western blot. Data Information: Panels (**A**) and (**B**) represent two independent experiments conducted similarly to those in Figs. 4B and 4D, respectively. Panel (**C**) shows the only co-immunoprecipitation of endogenous Bri2 and Trem2 performed to date. The complete membrane images used for Western blot analyses are included without any cropping of information above or below the targeted signals.

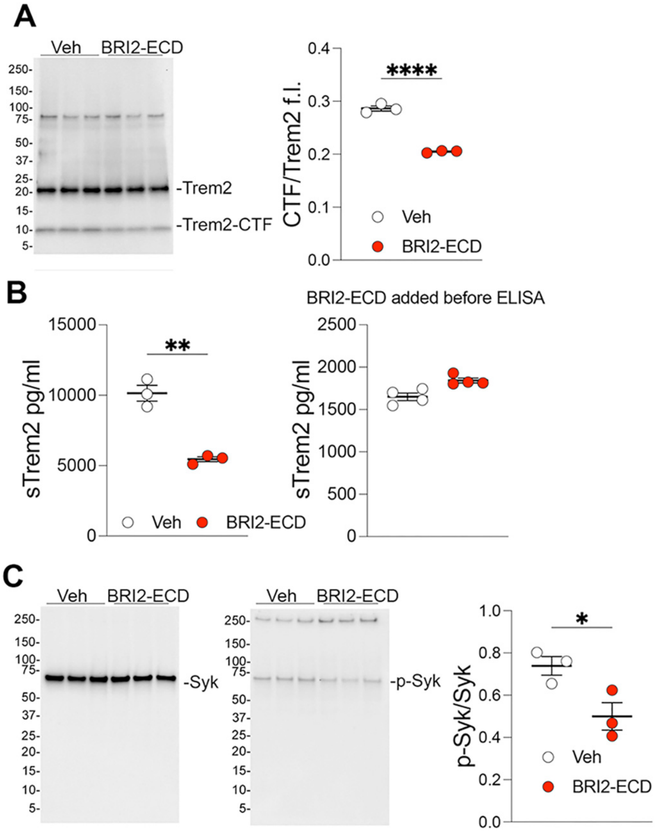

Figure EV4. Evidence of the effect BRI2-ECD on Trem2 processing and signaling.

(**A**) Western blot analysis with Trem2 CT antibody of deglycosylated cell lysates from *Itm2b-KO* microglia treated with either vehicle (PBS) or a 2 μM concentration of BRI2-ECD. Quantification of the Trem2-CTF/Trem2 f.l. ratios from the Western blot shown in the right panel. (**B**) sTrem2 ELISA on conditioned media from these cell cultures (left panel). The right panel shows an ELISA performed using media from cells treated with vehicle and incubated before and during the ELISA with either vehicle (PBS) or 2 μM of BRI2-ECD. The evidence that incubation with BRI2-ECD does not change the ELISA quantification indicates that BRI2-ECD does not interfere with the quantification of sTrem2 by ELISA. (**C**) Western blot analysis with anti-Syk and anti-pSyk antibodies of cell lysates from *Itm2b-KO* microglia treated with either vehicle (PBS) or a 2 μM concentration of BRI2-ECD. Quantification of the pSyk/Syk ratios from the Western blot is shown in the right panel. Data information: This figure encompasses the comprehensive dataset employed for these specific experiments. We have included the images of the complete membranes used for Western blot analyses, without any cropping of information above or below the targeted signals. Statistical comparisons among the groups were conducted using a two-tailed unpaired *t*-test. *$P < 0.05$, **$P < 0.01$, ****$P < 0.0001$. The data presented are derived from *Itm2b-KO* primary microglia ($n = 3$ for each condition); the letter "n" indicates biological replicates. Each biological replicate was composed of primary microglia generated from 2 P2 pups. All data are expressed as means +/− SEM. Source data are available online for this figure.

 © The Author(s)

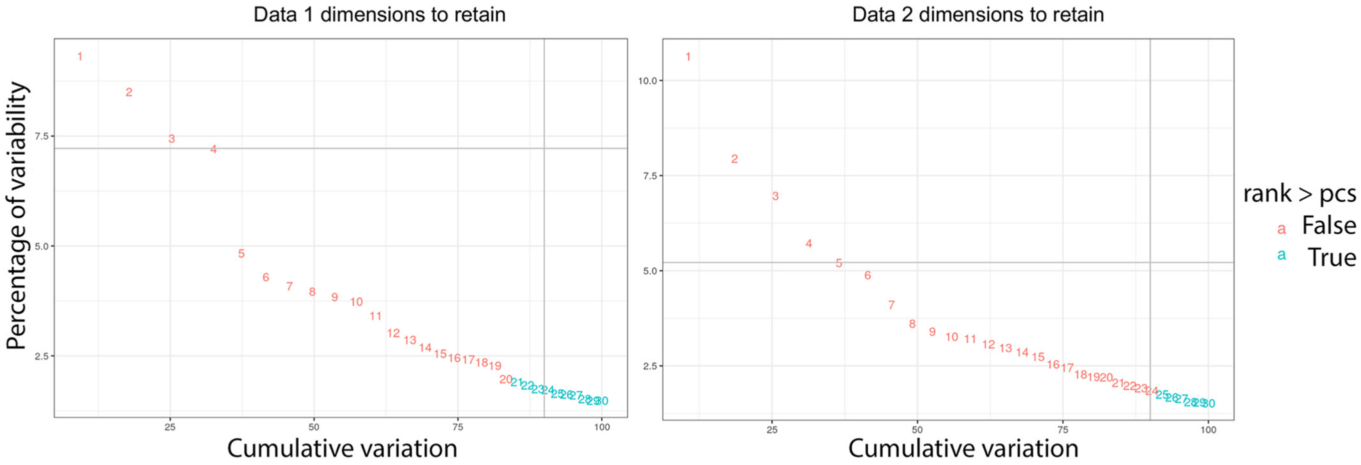

Figure EV5. Heuristic establishment of the number of dimensions/principal components (PCs) needed to capture 90% of the variance in gene expression from the datasets.

© The Author(s)
